# *Brucella abortus* egresses from host cells in infective clusters through an actin-dependent mechanism

**DOI:** 10.1128/msphere.00450-26

**Published:** 2026-09-24

**Authors:** José Fabio Campos-Godínez, Alejandro Hernández-Saborío, Tiffany Vargas-Moya, Valeria Gómez-Vargas, David Espinoza-Villagra, Ignacio Sandoval, María Paula Rojas-Salas, Melany López-Hernández, Rodrigo Vega-Arce, Reynaldo Pereira-Reyes, Monica Prado, Carlos Chacón-Díaz, Edgardo Moreno, Esteban Chaves-Olarte, Pamela Altamirano-Silva

**Affiliations:** 1 Centro de Investigación en Enfermedades Tropicales, Facultad de Microbiología, Universidad de Costa Rica 27915, San José, Costa Rica; 2 Centro Nacional de Alta Tecnología, Pavas, San José, Costa Rica; 3 Centro de Investigación en Ciencia e Ingeniería de Materiales (CICIMA), San José, Costa Rica; 4 Programa de Investigación en Enfermedades Tropicales, Escuela de Medicina Veterinaria, Universidad Nacional 504365, Heredia, Costa Rica; Virginia-Maryland College of Veterinary Medicine, Blacksburg, Virginia, USA

**Keywords:** egress, intracellular life, clusters, actin mechanism, intracellular pathogen, intracellular life cycle, infective

## Abstract

*Brucella abortus* is an intracellular pathogen whose cell cycle encompasses attachment, internalization, trafficking, replication, and egress from host cells. Although the intracellular life of *Brucella* has been extensively studied, the mechanisms underlying its exit from host cells remain unclear. In this work, we expand the knowledge of this intracellular step by observing a significant increase in the formation of *Brucella*-containing vacuoles with autophagic features (aBCVs) and abundant *B. abortus* extracellular clusters (BECs) after 72 h of infection. Membrane extensions protruding through actin polymerization were evident in cells at later stages of infection. Purified aBCVs and BECs were similar in size and predominantly acidic. These vacuoles exhibited a compact arrangement of well-ordered bacteria, comprising a heterogeneous population of dead and live bacteria and host components, including LAMP-1 and actin filaments. A proportion of BECs were enclosed within an impermeable host membrane, while others were not. The actin cytoskeleton was implicated in the protrusion of BECs, since modulation of Rho GTPases affected intracellular aBCV formation and the egress of BECs. BECs protruding from cells invaded uninfected cells, initiating a new cycle of infection. The topological structure and function of BECs underscore their significance in the *Brucella* life cycle as vehicles for bacterial dissemination to host cells and organs.

**IMPORTANCE:**

*B. abortus* is an intracellular pathogen that traffics to the endoplasmic reticulum, where it replicates. However, the mechanisms by which *Brucella* exits host cells and infects new ones remain poorly understood. Our findings reveal that protruding acidic autophagic-like vesicles containing compact, well-organized *Brucella* clusters are shed from cells via the GTPase-dependent recruitment of actin filaments. Through this process, the vesicles are impermeable to antibodies and other substances, like antibiotics, and are highly infectious to neighboring cells. This mechanism enhances the understanding of the well-known *Brucella* stealth strategy to evade the immune system and establish harmful, long-lasting infections.

## INTRODUCTION

*Brucella abortus* is a gram-negative, intracellular, facultative-extracellular bacterium that causes a persistent brucellosis disease (1). In cattle, *B. abortus* induces abortions, low milk production, orchitis, epididymitis, and infertility. In humans, *B. abortus* transmission occurs through direct contact with infected animals or their contaminated products, resulting in a debilitating disease that can be fatal if untreated (2).

Antibody- or complement-opsonized *B. abortus* can be internalized through zipper phagocytosis by macrophages and monocytes. Alternatively, non-opsonized *Brucella* can enter via fibronectin and lectin receptors or through alternative mechanisms that are not yet fully elucidated (3). Epithelial cells internalize *Brucella* by recruiting actin filaments and activating small GTPases such as Cdc42, Rac, and Rho (3, 4). Inside phagocytic or non-phagocytic cells, *Brucella* is contained within a membrane-enclosed compartment known as the *Brucella*-containing vacuole (BCV) (5, 6).

The BCV interacts with early and late endosomes, acidifying the compartment to ~pH 4.5 (7), thereby transitioning to an early acidified endosomal BCV (eBCV) (8). The eBCV then progressively loses endosomal markers while interacting with endoplasmic reticulum exit sites, acquiring markers such as calreticulin, calnexin, and sec61β. At this stage, *Brucella* organisms initiate replication (rBCV) (8). Following extensive bacterial replication, the rBCV is captured within acidic autophagosome-like structures, forming an autophagic BCV (aBCV) (8). At later stages of infection, *B. abortus* sequesters multivesicular bodies (9), which are generated by invagination and budding of the limiting membrane (10). Multivesicular bodies can fuse with autophagosomes, forming vesicles called amphisomes, which subsequently fuse with the plasma membrane, thereby allowing bacterial egress in multicellular organisms (9). To support completion of its intracellular cycle and egress from both HeLa cells and macrophages, *Brucella* modulates host iron metabolism, mitochondrial fragmentation, and BNIP3L-mediated mitophagy (11, 12).

Cells heavily infected with *Brucella* can divide without clear signs of cytotoxic effects (13), and some cells, such as trophoblasts, may show large acidic vacuoles containing clusters of bacteria that display LAMP-1 markers (14). In this study, we demonstrate that as aBCV, the purified BCV extracellular clusters (BECs) released from infected cells are characterized by low pH, LAMP-1, and actin filament markers. While a membrane surrounds some BECs protruding from the cell membrane, others are “naked” and released from host cells via actin filaments and small GTPases of the Rho subfamily. The released BECs successfully infect new host cells, promoting the dispersion of *Brucella* and likely evading the immune system.

## RESULTS

### *B. abortus* exit from host cells over time is dynamic

**Fig 1:**
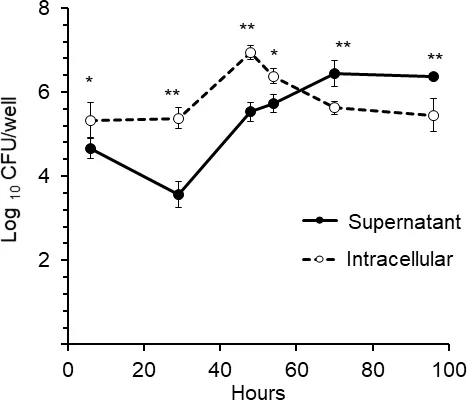
*B. abortus* exit from host cells is a dynamic process. HeLa cells were infected with *B. abortus* 2308W in the early exponential phase. At the indicated times, the infected cells were washed and then incubated for 4 h in DMEM without gentamicin. Bacteria from the supernatant were collected and plated, and CFU counts were performed. Then, cells were washed with PBS and lysed with 0.1% Triton, and intracellular CFUs were determined. Each value is the average of at least three independent determinations. *P < 0.05, **P < 0.005.

HeLa cells were infected with *B. abortus* 2308W, and the number of intracellular and extracellular bacteria was determined over time (Fig. 1). *B. abortus* exit is a dynamic process increased by bacterial replication, with its maximum estimated time reached at 72 h post-infection (p.i.).

### *B. abortus* arranges in two different replicative patterns within cells

**Fig 2:**
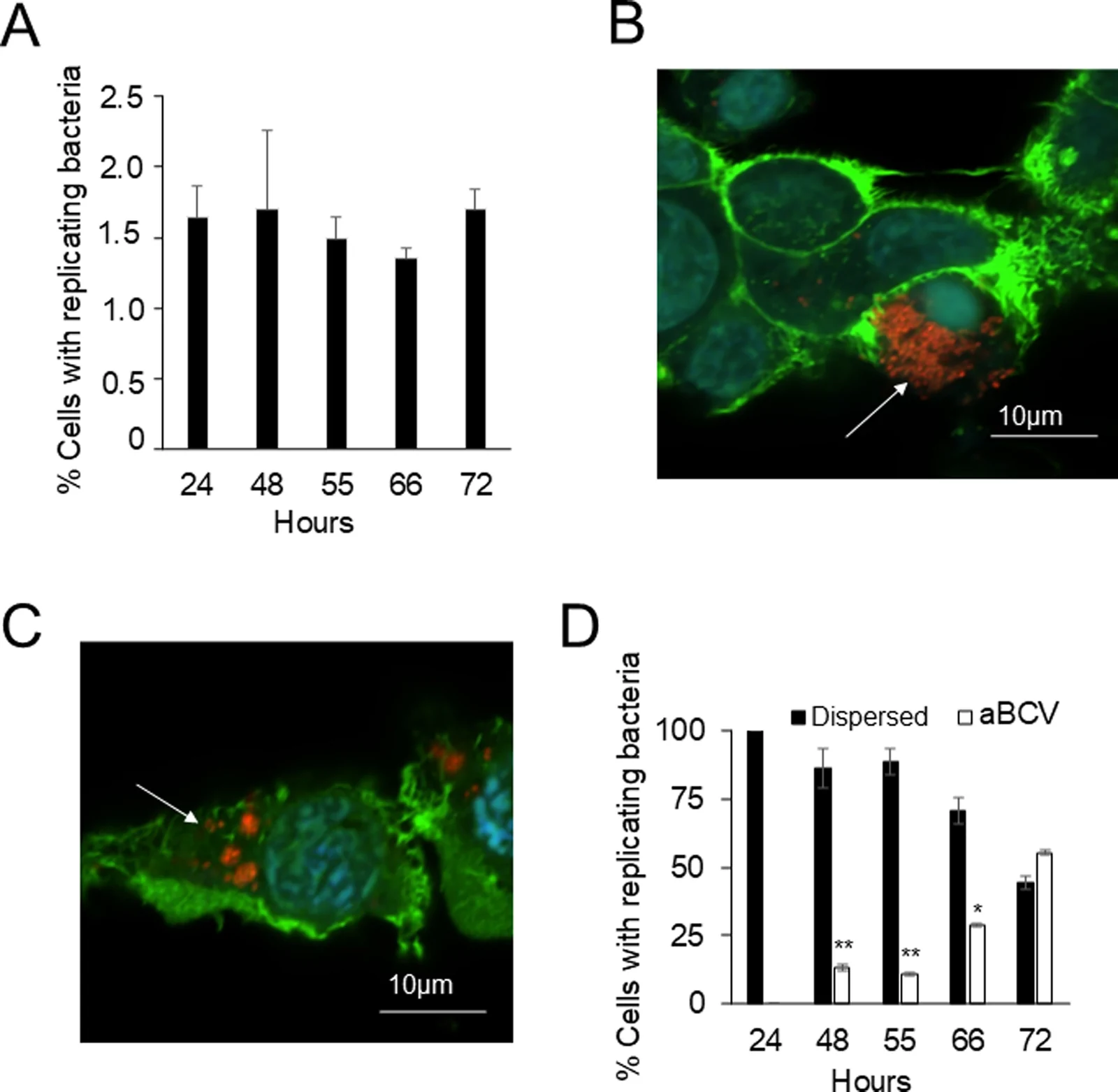
*B. abortus* within intracellular and extracellular vesicles. HeLa cells were infected with an exponential-phase culture of *B. abortus-*RFP (multiplicity of infection of 500). At the indicated times, cells were fixed with paraformaldehyde, and their nuclei were stained with DAPI (blue) and visualized by fluorescence microscopy. (**A**) Proportion of cells with replicating bacteria (>10 bacteria/cell). (**B**) Immunofluorescence of cells with replicating bacteria dispersed throughout the cytoplasm (white arrow). (**C**) Immunofluorescence of cells with aBCVs (white arrow). (**D**) Proportion of cells with dispersed bacteria and aBCVs. Each value is the average of at least three independent determinations. **P < 0.005, *P < 0.05.

HeLa cells were infected with *B. abortus*-red fluorescent protein (RFP), and bacteria and infected cells were visualized by fluorescence microscopy over time. No significant differences in the proportion of HeLa cells with replicating *B. abortus* were observed between 24 and 72 h p.i. (Fig. 2A). However, two distinct replicative patterns were noted: (i) infected cells with bacteria homogeneously dispersed throughout the cytoplasm (Fig. 2B) and (ii) cells with intracellular bacterial clusters previously named aBCV (Fig. 2C). From 24 to 66 h p.i., 70%–100% of the cells containing replicating bacteria exhibited dispersed bacteria (Fig. 2D). Conversely, a significant increase of aBCVs was observed in cells after 72 h p.i. Approximately 56% of the infected cells with replicating bacteria exhibited aBCVs at 72 h p.i. (Fig. 2C and D).

### *B. abortus* organisms protrude from infected cells at later stages of infection

**Fig 3:**
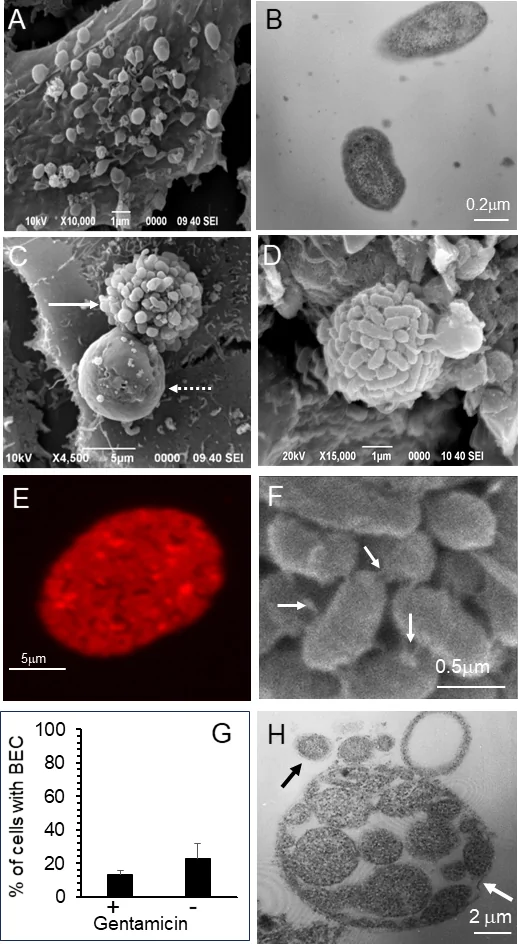
*B. abortus* protrude from cells as single bacteria, naked BECs, or membrane-enclosed BECs. HeLa cells were infected with an exponential-phase culture of *B. abortus*-RFP (multiplicity of infection of 500). After 72 h, cells were incubated without antibiotics for 4 h, fixed, and processed for scanning or transmission electron microscopy or fluorescence microscopy. (**A**) Scanning electron microscopy of individual bacteria protruding from infected HeLa cells. (**B**) Transmission electron microscopy of single bacteria released by infected HeLa cells. (**C**) Scanning electron microscopy of two forms of BEC protrusion: “naked” BEC (continuous white arrow) or membrane-enclosed BEC (discontinuous white arrow). (**D**) Protruding naked BECs are arranged in tightly packed aggregates of bacteria. (**E**) Fluorescent BEC filled with *B. abortus*-RFP secreted by an infected cell after 100 h of infection. (**F**) Interbacterial linking structures within the bacterial aggregates (white arrows). (**G**) From 48 to 100 h p.i., infected cells were monitored by live imaging, and the proportion of BECs protruding from infected cells was determined in the presence or absence of gentamicin. (**H**) Transmission electron microscopy of purified aBCV. Notice the thin cell-derived membrane surrounding the aBCV (white arrow) and a free bacterium (black arrow).

HeLa cells were infected with *B. abortus*-RFP, at later times of infection, *B. abortus* protrudes from infected cells as single organisms or as BECs of different sizes (Fig. 3A through D). The protruding BECs of bacteria may be naked or surrounded by a host membrane structure (Fig. 3C). Scanning electron microscopy revealed that bacteria in BECs are arranged in closely packed, organized clumps (Fig. 3C through E) interlinked by thin structures (Fig. 3F). Furthermore, to rule out that these structures are host cell-derived vesicles, we performed comparative observations in non-infected cells, where these specific structures were consistently absent. The proportion of infected cells with BECs protruding after 80–83 h p.i. was close to 20%. Egress was not significantly affected by the presence of gentamicin in the culture media, suggesting that some of the BECs originating from aBCVs had a protective membrane derived from the host cell (Fig. 3G), a phenomenon confirmed by electron microscopy (Fig. 3H).

Historically, the HeLa cell model has been extensively utilized in cellular microbiology with several pathogenic bacteria, including *Brucella*, to validate infection, intracellular trafficking, and protruding mechanisms, primarily due to the optimal characteristics of these cells for high-resolution imaging (8, 9). Moreover, epithelial HeLa cells serve as a model for other target cells, such as epithelial trophoblasts. However, to ensure that our observations were not unique to the epithelial cell model, we replicated our experiments in macrophages. The observation of BECs in macrophages (Fig S1) reinforces the biological relevance of this process and allows us to deepen the characterization of the previously described *Brucella* egress (9).

### A host-derived membrane surrounds a significant proportion of BECs and aBCVs

**Fig 4:**
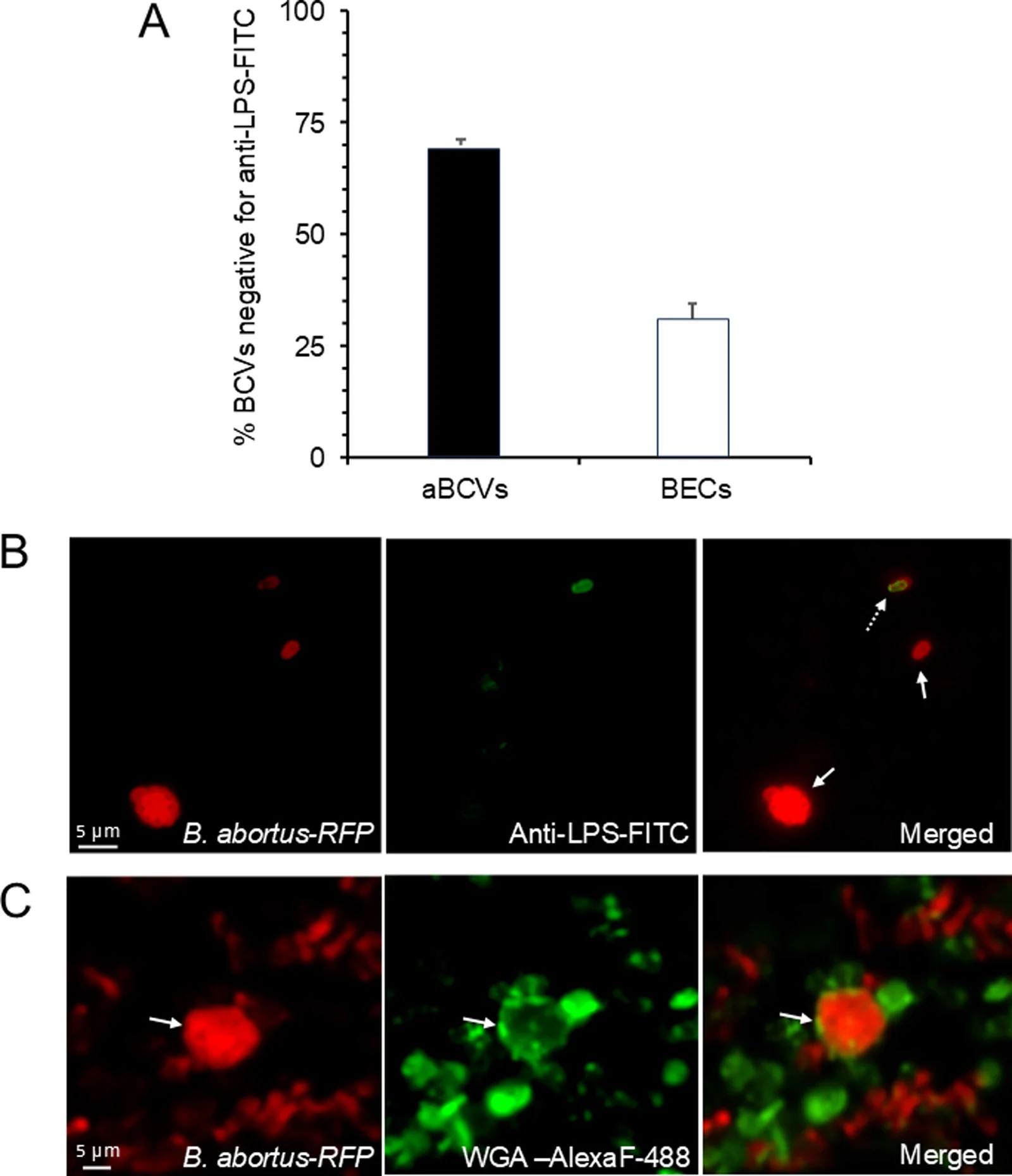
The membrane-surrounded BECs and aBCVs are impermeable to antibodies. HeLa cells were infected with an exponential-phase culture of *B. abortus*-RFP (multiplicity of infection of 500). After 72 h of incubation, cells were incubated without antibiotics for 4 h, and the supernatant was collected. The cells were then lysed, and aBCV and BECs were enriched by centrifugation cycles and stained with fluorescent conjugates. (**A**) Purified aBCVs and BECs were stained with anti-*Brucella* LPS-FITC without permeabilization and counted. Each value is the average of at least three independent determinations. (**B**) Purified aBCVs stained with anti-*B*. *abortus*-LPS-FITC. The discontinuous arrow points to a bacterium not surrounded by an impermeable host cell membrane, while the continuous arrow points to aBCVs and a bacterium impermeable to anti-*B*. *abortus*-LPS-FITC. (**C**) Purified BECs were stained with wheat germ agglutinin-Alexa Fluor 488 (WGA-Alexa Fluor 488, green). White continuous arrows indicate the BECs surrounded by an impermeable membrane. The contrast was adjusted to no more than 20% in the same proportion across the respective images using the hue adjustment function in Adobe Photoshop. Raw images were processed using the denoise.ai module.

Analysis of the supernatants of *B. abortus*-RFP HeLa-infected cells shows the release of individual bacteria and BECs. In each 60× field examined, ~1,000 free bacteria and at least 1 BEC were present. To determine the proportion of BECs covered by a host-derived membrane, purified vesicles were incubated with anti-*Brucella* LPS-fluorescein isothiocyanate (FITC) antibody under non-permeabilizing conditions. Approximately 70% of the BECs were stained with anti-*Brucella*-LPS antibodies, indicating that the remaining ~30% were surrounded by a host-impermeable membrane (Fig. 4A and B). In addition, a significant proportion of purified aBCVs was also covered by a host-derived membrane (Fig. 4A). To corroborate these findings, the BECs were labeled with wheat germ agglutinin conjugated with Alexa Fluor 488, which primarily reacts with N-acetyl-D-glucosamine and sialic acid residues of glycoproteins on cell membranes (Fig. 4C). Overall, the results indicate that the BECs are a heterogeneous population of vesicles, with approximately one-third of them surrounded by an antibody-impermeable membrane. Those BECs permeable to antibodies may still be partially surrounded or contain disrupted membranes.

To explore whether non-stained BECs under non-permeabilizing conditions were not a consequence of lipopolysaccharide (S-LPS) O-antigen dissociation or a transition to a rough phenotype, we performed validation experiments on extracted BECs. First, lysis of the enriched BECs to obtain contained bacteria, followed by immunofluorescence and western blotting, confirmed that recovered bacteria remained positive for the O-antigen (Fig S2A and B). The characteristic ladder-like pattern of S-LPS remains consistent, indicating that the O-antigen is retained and not dissociated or shed during the intracellular cycle. Additionally, 100% of purified bacteria were stained by immunofluorescence with anti-*Brucella* LPS-FITC antibody. Furthermore, permeabilization of enriched BECs with 0.1% Triton resulted in 100% labeling efficiency, with all internal bacteria becoming strongly immunoreactive to anti-*Brucella* antibodies (Fig S2C). Collectively, these results and labeled BECs with wheat germ agglutinin confirm that the observed lack of staining in intact clusters is due to the physical barrier of an antibody-impermeable membrane rather than loss of surface antigenic determinants of S-LPS.

### BEC and aBCV size and shape

**Fig 5:**
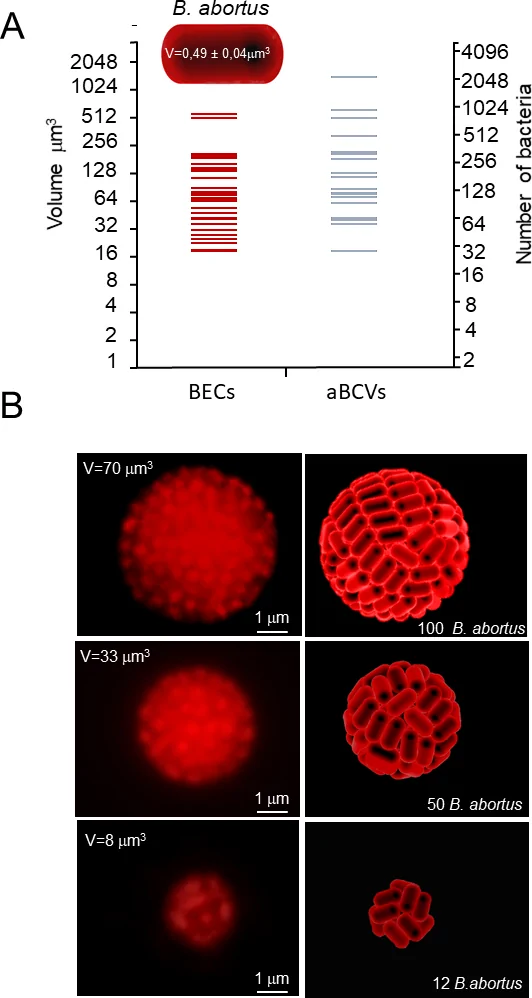
Structural arrangement of BECs and aBCVs. Enriched aBCVs and BECs were counted and observed under a confocal fluorescence microscope. (**A**) Volumes of BECs and aBCVs, and the number of *B. abortus*-RFP per vacuole. The *B. abortus* volume estimation was 0.49 ± 0.04 µm³ (inserted model). (**B**) BECs of different sizes and the corresponding packing and number of *B. abortus*-RFP per BEC were estimated for a given volume. The photographs of the vacuoles were amplified (left panels); the number of individual *B. abortus*-RFP was calculated using the Adobe Photoshop density color pixel function of the image’s sections, and the packing of bacteria (*l*/*d* = 1) within vacuoles modeled (right panels) following the method for close packing of short rods within spherical surfaces (16). The contrast was adjusted to no more than 20% in the same proportion in the respective images using the hue adjustment function of Adobe Photoshop.

The average volume of an uninfected HeLa cell ranges from 2,500 to 3,000 μm^3^, a volume that may increase in infected cells containing *Brucella* organisms (13, 15). Consequently, infected cells containing a significant number of aBCVs may release BECs of various sizes, each containing a variable number of bacteria. The immunofluorescence analysis of BECs and aBCVs revealed that these vesicles may accommodate ~10 to ~650 bacteria (Fig. 5A). The more abundant BECs and aBCVs carry fewer than 300 bacteria. Vacuoles above 250 μm^3^ are significantly less abundant than smaller ones and probably more prone to disruption due to lower pressure and a larger surface area-to-volume ratio.

Amplification of BEC photographs of various sizes revealed a regular arrangement of bacteria within the vacuoles (Fig. 5B). This action allowed their analysis by color density differences through the topology of the BEC photographs and the estimation of the approximate number of bacteria within the vacuoles. Following the estimation method for the close packing of short rods within spherical surfaces (16), we modeled the packing of *B. abortus* coccobacilli within BECs of three different sizes (Fig. 5B). The arrangement of coccobacilli in each of the three BECs differed considerably. For the structures with 50 and 100 coccobacilli, the spherical arrangements were consistent with the BECs photographs, as predicted by the theory for packing short rods. However, for the smaller BECs, pairs of nearly parallel coccobacilli forming the faces of a cube were observed (Fig. 5B).

### BECs and aBCVs contain a heterogeneous population of *B. abortus*

**Fig 6:**
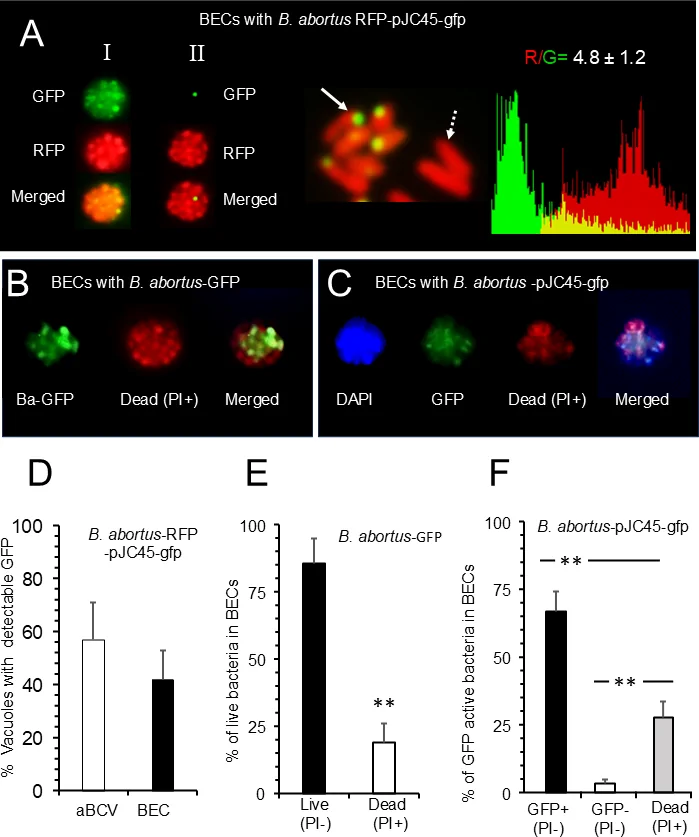
BECs and aBCVs have a heterogeneous population of *B. abortus*. HeLa cells were infected with an early-exponential-phase inoculum of either Ba-GFP (*B. abortus*-GFP), *B. abortus-*pJC45-gfp, *or B. abortus*-RFP-pJC45-gfp (multiplicity of infection of 500). After 72 h, the cells were incubated without antibiotics for 4 h, and aBCVs and BECs were enriched and analyzed. (**A**) To the left of the panel: a BEC containing *B. abortus*-RFP-pJC45-gfp with practically all bacteria expressing GFP (I), and a BEC containing only one bacterium expressing GFP (II). In the center of the panel: *B. abortus*-RFP-pJC45-gfp expressing GFP (unbroken arrow) and live bacteria non-expressing GFP (broken arrow). To the right, a spectrum of one bacterium expressing GFP (unbroken arrow). Based on the analysis of 50 spectra, the ratio between red and green (R/G) fluorescence was estimated. We calculated the R/G ratio, a mathematical normalization to accurately evaluate the relative distribution of both proteins and to prevent the overestimation of the RFP signal in the merged images. This approach allows for a more objective interpretation of the spatial relationship between the two markers, regardless of their individual brightness levels. (**B**) BEC containing live *B. abortus*-GFP (Ba-GFP) and dead *B. abortus*-GFP (PI+) organisms. (**C**) BEC with live, metabolically active, and dead *B. abortus* (DAPI in blue). In green, the metabolically active *B. abortus*-pJC45-gfp expressing GFP. In red are dead bacteria stained with propidium iodide (PI+). (**D**) Proportion of BECs and aBCVs with at least one metabolically active *B. abortus*-RFP-pJC45-gfp expressing GFP. (**E**) Enriched BECs containing *B. abortus*-GFP were lysed with 0.1% Triton, and the bacterial viability was determined using propidium iodide (PI) stain. (**F**) Enriched BECs containing *B. abortus-*pJC45-gfp were lysed with 0.1% Triton, and the metabolic activity of bacteria was determined by inducing the production of GFP in *B. abortus*-pJC45-gfp through the action of anhydrotetracycline (250 nM) for 6 h. Then, all bacteria were visualized with DAPI, and dead bacteria were detected with PI. The contrast was adjusted to no more than 20% in the same proportion in the respective images using the hue adjustment function of Adobe Photoshop. **P < 0.005.

Most bacteria constitutively expressing fluorescent proteins, such as RFP or GFP, maintain cell integrity and are generally considered alive, in contrast to dead bacteria, whose fluorescence rapidly decays (17). The fluorescence analysis of BECs revealed heterogeneity in bacterial protein metabolism and in the living *B. abortus* population (Fig. 6A through C). The proportion of detectable *B. abortus*-RFP-pJC45-gfp displaying an inducible GFP (synonymous with metabolically protein synthesis-active bacteria) within BECs and aBCVs was not significantly different (Fig. 6D). The fact that close to 50% of the enriched vacuoles do not display detectable GFP does not mean that they are composed of just metabolically inactive bacteria but that the number of active bacteria present within them may be below the threshold of detection and that the expression of the GFP was not fully accomplished by the anhydrotetracycline (ATc) not readily diffusing inside the vacuoles (Fig. 6A). To prevent impaired ATC diffusion within the BECs from inhibiting GFP induction, BECs were enriched and lysed to isolate intracellular bacteria for subsequent analysis. Indeed, the proportion of live bacteria within BECs was close to ~82%, while the dead were ~18% (Fig. 6E). Likewise, the analysis of metabolically active and living inactive *B. abortus*, as well as dead bacteria, confirmed that BECs are composed of these three bacterial populations in different proportions (Fig. 6F). Altogether, these results indicate that BECs contain ~80% living organisms and ~20% dead bacteria; among the living organisms, close to 5% are not metabolically active. Notably, no double-positive (GFP+/PI+) bacteria were detected, as the GFP signal (indicative of metabolic activity) was absent in cells with compromised membranes labeled by PI.

### BECs and aBCVs are acidic and harbor LAMP-1

**Fig 7:**
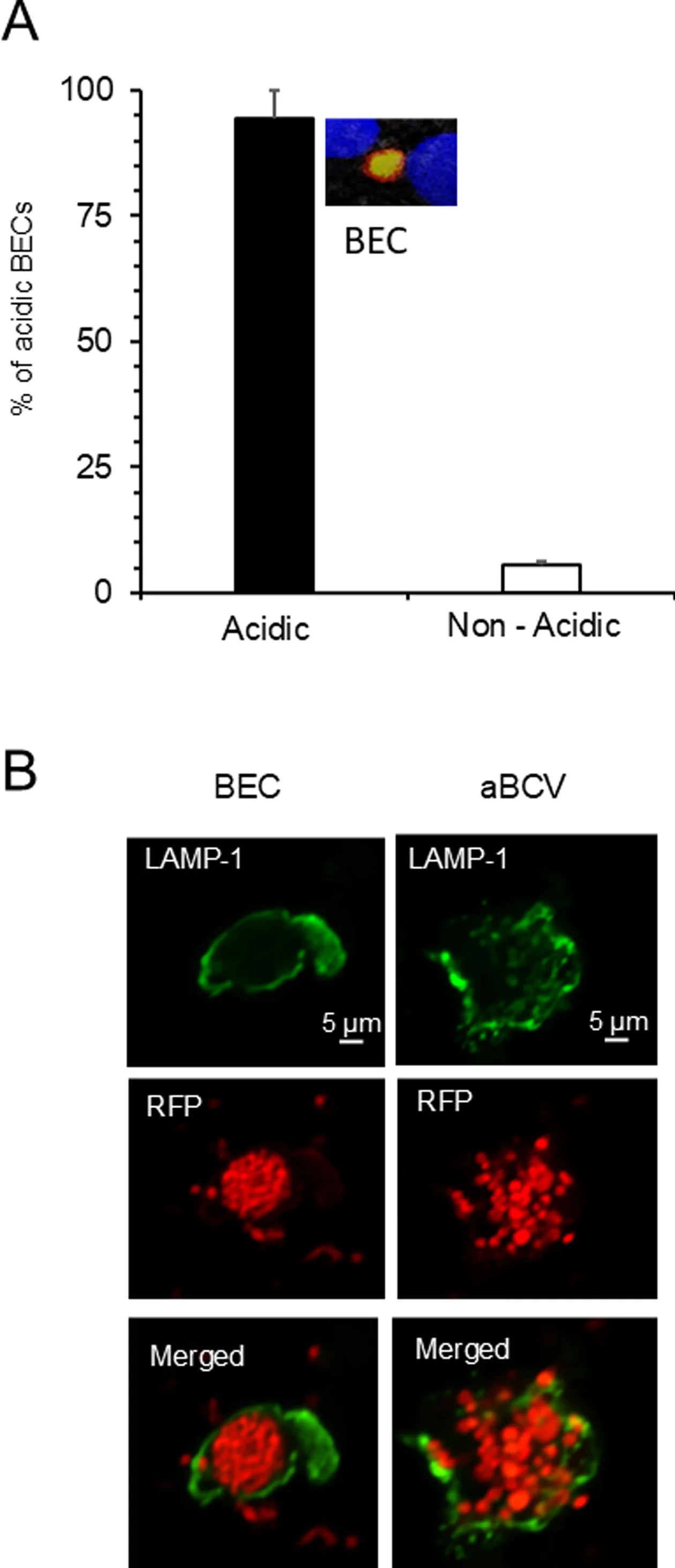
aBCVs and BECs are acidic and rich in LAMP-1. (**A**) Protruding BECs were labeled with 50 nM LysoTracker Red DND-99 (Invitrogen) for 1 h and observed through the BioTek Lion Heart Imager every 3 h until 100 h p.i., and the acidic BECs were counted. The insert shows a BEC in yellow. (**B**) Purified BECs and aBCVs were stained with anti-LAMP1-FITC and observed under the confocal laser fluorescent microscope. Raw images were processed using the Denoise.ai module.

Consistent with the previously described acidic properties of aBCV (8) and the similar proportion of aBCVs and BECs with *B. abortus* expressing GFP (Fig. 6D), we determined that close to 100% of BECs were acidic (Fig. 7A). Likewise, all examined aBCVs and BECs were labeled with the membrane protein LAMP-1 (Fig. 7B). Our results indicate that BECs originate from aBCVs and that the general properties of BECs are maintained after protrusion from the cell.

### BEC protrusion is linked to the actin-cytoskeleton polymerization

**Fig 8:**
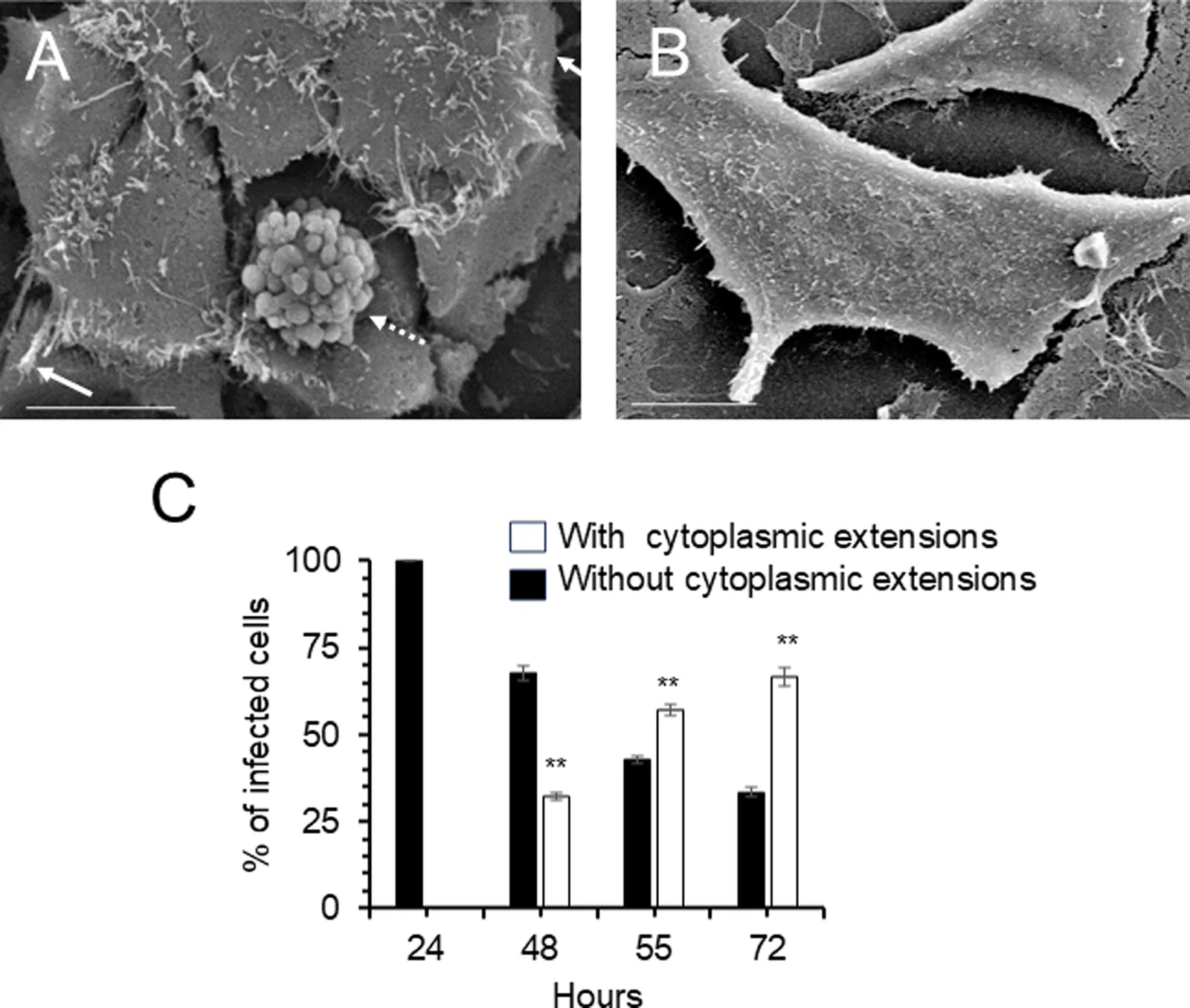
Cytoplasmic extensions are associated with protruding BECs. (**A**) Scanning electron microscopy of infected cells with protruding naked BECs (broken white arrow) and displaying long and numerous thin cytoplasmic extensions (unbroken white arrows). (**B**) Scanning electron microscopy of non-infected HeLa cells. (**C**) Proportion of infected HeLa cells with cytoplasmic extensions over time after infection. Each value is the average of at least three independent determinations. Statistical significance was assessed using the Kruskal-Wallis and Dunn’s multiple-comparison test (**, *P*  <  0.005 indicate significance from % cytoplasmic extensions at 24 h p.i.).

**Fig 9:**
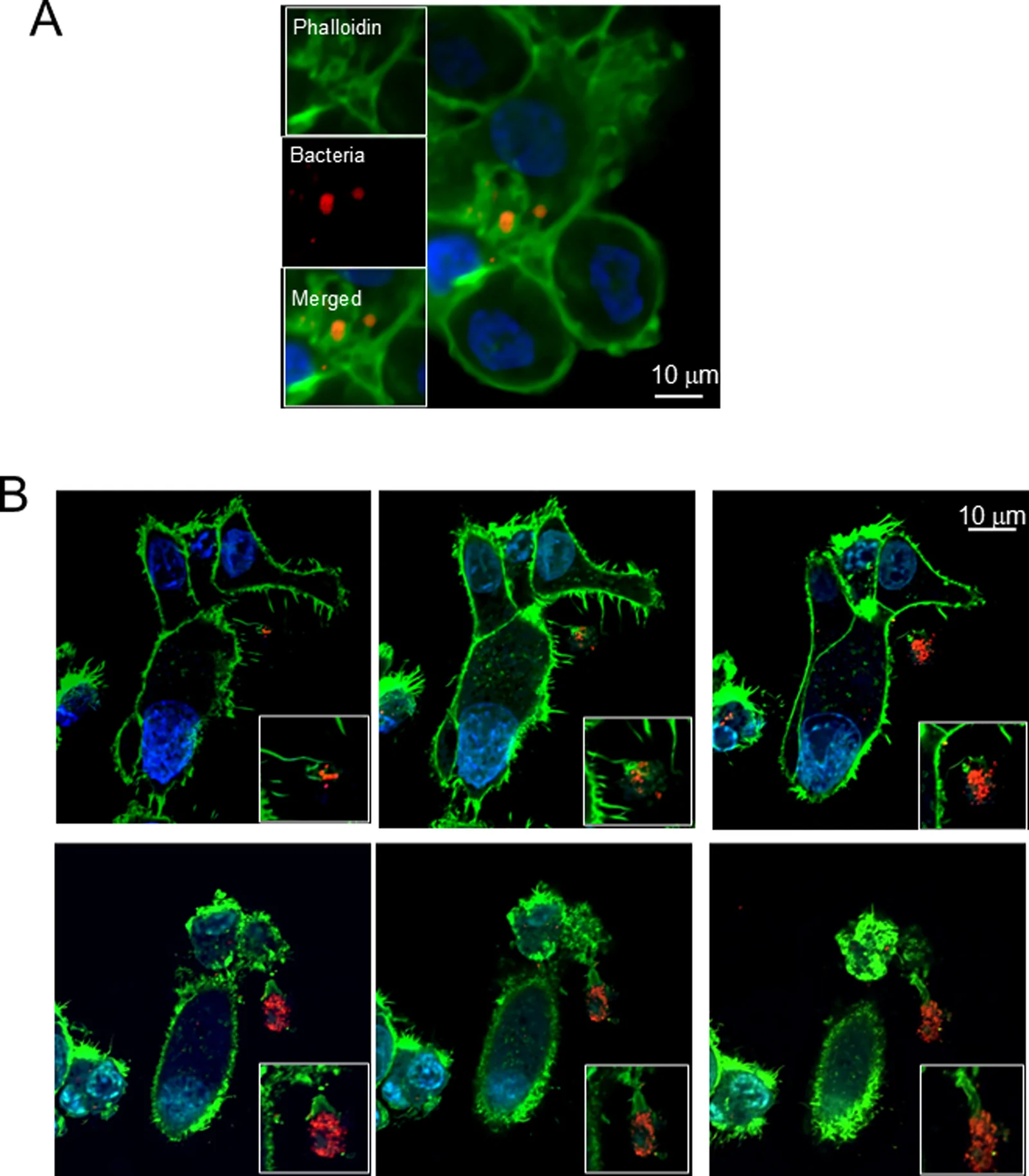
Actin filaments are associated with protruding BECs. HeLa cells were infected with an exponential-phase culture of *B. abortus*-RFP (multiplicity of infection of 500). At 72 h p.i., infected cells were fixed, stained with phalloidin-FITC, and observed under the fluorescent microscope. (**A**) HeLa-infected cell with intracellular aBCV stained with phalloidin (green). The insert is an amplification of an aBCV membrane stained with phalloidin. (**B**) Z-stack sequence of a BEC (in red) protruding from cells by actin cytoskeleton filaments stained with phalloidin (green). The cell nucleus was stained with DAPI (blue).

After 72 h p.i., single *B. abortus* and BECs were observed to emerge from infected cells in association with actin filaments (Fig. 8A). As the infection progressed, there was an increase in the proportion of infected cells with actin-mediated cytoplasmic extensions, which are notably shorter and less conspicuous in non-infected cells (Fig. 8B). To confirm that actin was associated with BECs protrusion, we labeled *B. abortus* RFP-infected HeLa cells with phalloidin. We observed that aBCV’s membrane in infected cells was labeled with phalloidin (Fig. 9A). Additionally, BECs emerge from infected cells in association with phalloidin-positive actin filaments (Fig. 9B). These data indicate an association between actin filament-mediated cell extensions and the protrusion of BECs.

### GTPases of the Rho subfamily promote the formation of aBCVs and protrusion of BECs

**Fig 10:**
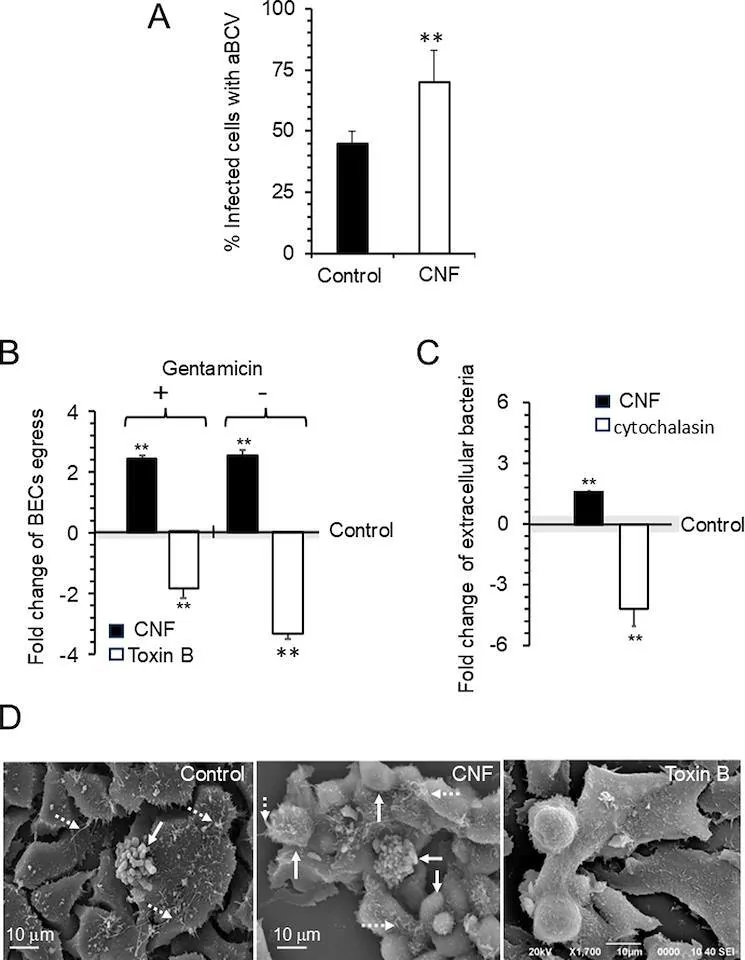
The aBCV exit is promoted by GTPases of the Rho subfamily and actin polymerization. HeLa cells were infected with a *B. abortus*-RFP (multiplicity of infection of 500) and received different treatments after 48 h post-infection. Then cells were incubated and observed, and data were collected after 72 h of infection. (**A**) Proportion of *B. abortus*-infected HeLa cells after treatment with CNF. (**B**) Fold increase and decrease in the protrusion of BECs from HeLa cells after treatment with CNF or *C. difficile* toxin B in the presence or absence of gentamicin. The standard error of the control is indicated by the gray area over the *X* axis. (**C**) Fold increase and decrease of extracellular bacteria with respect to the total number of bacteria (intracellular + extracellular) detected in infected HeLa cells treated with CNF or cytochalasin. The standard error of the control is indicated by the gray area over the “0” of the *X* axis. (**D**) Scanning electron microscopy of a *B. abortus*-infected HeLa cell without treatment (control), treated with CNF, and treated with *C. difficile* toxin B. Cells were observed and data collected after 72 h of infection. Notice that the protruding BECs (unbroken arrow) are accompanied by numerous long and thin cytoplasmic cell extensions (broken arrows) in the infected HeLa cells, which are more conspicuous in the CNF-treated cells, and practically absent in the toxin B-treated infected HeLa cells. Notice that the CNF-treated infected HeLa cells show a significant increase in the formation of naked and membrane-bound BECs. **P < 0.005.

Small GTPases of the Rho subfamily are involved in the mechanism of entrance of *B. abortus* to non-professional phagocytes (4). Because the *Escherichia coli* cytotoxic necrotizing factor (CNF) can permanently activate several of these GTPases (18), we evaluated the association of *B. abortus* to form aBCVs with GTPase activation by incubating infected HeLa cells with CNF. The activation of monomeric GTPases through the addition of CNF significantly enhanced the formation of aBCVs at later times of infection (Fig. 10A). Likewise, CNF also promoted the protrusion of BECs from the cells (Fig. 10B). In contrast, inhibition of Rho GTPases with *C. difficile* toxin B significantly decreased the BEC protrusion from the cells (Fig. 10B). These outcomes were consistent regardless of whether the infected cells were incubated with or without gentamicin. Moreover, exposure of infected cells to CNF significantly increases the bacterial release. In contrast, exposure to cytochalasin B inhibits bacterial release due to the disruption of the actin cytoskeleton (Fig. 10C). Scanning electron microscopy confirmed that CNF, in addition to promoting the protrusion of BECs, also favors the increase of membrane filaments, while toxin B works in the opposite direction, inhibiting the protrusion of BECs and actin-mediated membrane filaments (Fig. 10D). These events demonstrate that recruitment of GTPases and the concomitant actin polymerization are necessary events for the efficient generation of aBCVs and protrusion of bacteria and BECs.

### BECs contain infective brucellae

**Fig 11:**
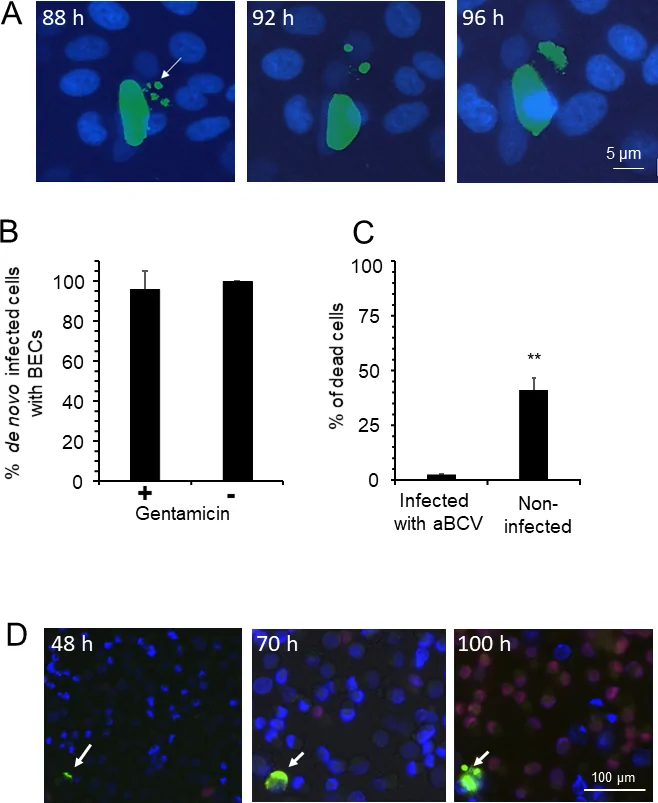
BECs shed by HeLa cells initiate a new cycle of infection. HeLa cells were infected with *B. abortus*-GFP (multiplicity of infection of 500). After 48 h, cells were washed with PBS and incubated with or without gentamicin or propidium iodide. (**A**) BEC shedding by an infected HeLa cell (white arrows) and *de novo* infection of uninfected cells were monitored using live imaging every 3 h for 100 h. *B. abortus*-GFP is shown in green, while cell nuclei are shown in blue. The frames are the same microscopic field at different times. (**B**) The proportion of BECs that infect new cells and initiate replication was determined in the presence or absence of gentamicin. (**C**) Proportion of dead cells in non-infected and infected HeLa cells with protruding BECs. (**D**) Fluorescent microscopy of *Brucella*-GFP-infected and non-infected HeLa cells stained with propidium iodide at different times. The *B. abortus*-GFP-infected cell (white arrow) remains alive (not stained with propidium iodide) and in time shedding BECs, while a large proportion of non-infected cells died after 100 h of culture (stained in red). The frames are the same microscopic field at different times. Each value is the average of at least three independent determinations. No significant differences (*P*  <  0.05) were found after treatment with or without gentamicin. Statistical significance was assessed using the Kruskal-Wallis test and Dunn’s multiple-comparison test ( **, *P*  <  0.005).

To determine whether *B. abortus* organisms inside BECs can infect cells, live imaging was conducted 48–100 h p.i. (Fig. 11A). Remarkably, close to 100% of the BECs initiated a new infection cycle regardless of the presence or absence of gentamicin in the culture media (Fig. 11B and Video S1). Exposure to propidium iodide revealed that after more than 4 days of culture, the cell membrane integrity remained impermeable in close to 100% of the BECs donor-infected cells, in contrast to the non-infected cells. This event indicates that *B. abortus* intracellular proliferation and later BEC shedding prolong the life of these infected cells for a protracted period (Fig. 11C and D).

## DISCUSSION

Several strategies for the egress of intracellular bacterial pathogens from the host cells have been reported (5, 9, 19–22). These pathways include the release of single, naked bacteria following host cell death mediated by toxic bacterial mediators or the cell disruption caused by stress mechanisms due to overcrowding of bacteria within host cells. Alternatively, bacteria may egress by non-lytic mechanisms, either by actively breaching host cell-derived membranes or by being surrounded by host cell membranes. In this latter mechanism, bacteria may protrude as single cells or as large vesicles filled with bacteria (21). As expected, these mechanisms are not mutually exclusive and can be observed during a cell infection process with a given pathogen (21).

In *B. abortus*, it has been shown that heavily infected trophoblasts release free bacteria into the uterine lumen after cell necrosis and ulceration of the chorioallantoic membranes, a phenomenon associated with the overwhelming infection of these cells (5, 19). Disruption of heavily *Brucella*-infected cells does not result from direct toxic action by the replicating organisms but is likely due to metabolic exhaustion and stress (13, 15). Indeed, at later times of infection, *Brucella* organisms occupy most of the cytoplasmic space of the epithelial cells within endoplasmic reticulum-derived structures (5, 6, 13, 19, 23). Consequently, one alternative is that *Brucella* organisms trigger an endoplasmic reticulum stress response, which eventually disrupts the heavily infected cell, releasing free bacteria into the milieu (24).

In agreement with our previous observations, we demonstrate that infected HeLa cells shedding BEC survive longer than most non-infected cells in cultures. This event suggests that *Brucella* organisms arrest cell death of infected cells for a protracted period, allowing for high intracellular replication and the eventual shedding of highly infectious BECs. However, it seems plausible that after a prolonged time (>100 h), these heavily infected cells would exhaust and eventually die, releasing bacteria into the surrounding milieu, as previously indicated and observed in infected trophoblasts (5, 19).

**Fig 12:**
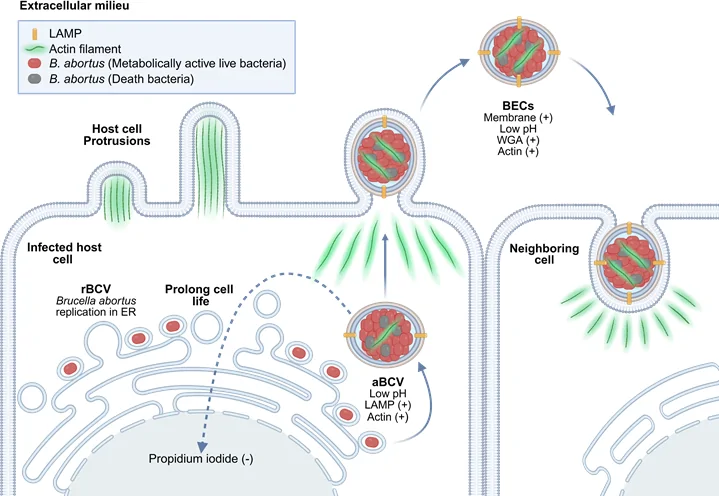
Conceptual model illustrating the dynamics of *B. abortus* release from previously infected cells**.** Following extensive replication in endoplasmic reticulum-derived compartments, *B. abortus* organisms assemble into an acidic vacuole with autophagosomal characteristics (aBCVs), as described by Starr et al. (8). During this period, replicating bacteria extend the lifespan of the infected cell. Dissemination through BECs. The BEC formation is initiated by the activation of Rho subfamily GTPases and the active polymerization of actin filaments.

*Brucella*-containing vacuoles acquire autophagic characteristics (24, 25), promoting a non-lytic exocytosis process through the sequestration of multivesicular bodies forming amphisomes that eventually fuse with the plasma membrane and generate bacterial egress as vesicles (9). Here, we validated this trafficking pattern and extended the findings on the dynamics, composition, and infectious properties of the BECs. A model of our proposal for the egress of *Brucella* organisms in BECs from infected cells is presented in Fig. 12. Following extensive replication in endoplasmic reticulum-derived compartments, *B. abortus* organisms assemble into an acidic vacuole with autophagosomal characteristics (aBCV) (8). During this period, replicating bacteria extend the lifespan of the infected cell. Then, the BEC formation is initiated by the activation of Rho subfamily GTPases and the active polymerization of actin filaments. The released BECs retain the acidic properties and general characteristics of aBCVs. The membrane may protect the bacteria from immune detection and facilitate infection of neighboring cells. Additionally, free *B. abortus* organisms are released into the extracellular milieu. These single bacteria can reach adjacent cells or distant sites, although they are more vulnerable to immune clearance. Infected cells display membrane protrusions, suggesting actin recruitment. This may allow the bacteria to spread while avoiding immune exposure. A notable observation in our study is the resistance of extracellular BECs to gentamicin, which contrasts with previous reports by Starr et al. (8) and Spera et al. (9), where egressed *Brucella* structures were described as antibiotic sensitive. We hypothesize that these differences may stem from the specific infection stage and the host cell model employed. In our study, at 72 h p.i., the egressed clusters are enclosed within a robust, antibody-impermeable membrane that also appears to provide a physical barrier against aminoglycoside penetration. The lack of significant differences in bacterial recovery between clusters treated with or without gentamicin further supports the functional integrity of this surrounding membrane. These findings suggest that *Brucella* may utilize these membrane-bound structures not only as a vehicle for egress but also as a protective niche that ensures survival in the extracellular environment before reaching a new host cell, a strategy that could be critical for the persistence of the infection in different tissue contexts. Our findings are further supported by Spera et al. (9), where the authors show that treatment with Triton X-100 significantly increases the number of gentamicin-sensitive bacteria. This corroborates our proposal that the bacteria within the clusters are protected by a membrane that must be chemically permeabilized for the drug to act.

We do not know why some infected cells primarily generate aBCVs, while others display dispersed bacteria throughout the cytoplasm, a phenomenon observed not only in cultured HeLa cells but also in other epithelial cells, such as placental trophoblasts (5, 14, 17). Certainly, the tendency to form large aBCVs increases over time, possibly related to the crowded effect within the cytoplasm. It is known that crowding can affect not only diffusion rates and macromolecular interactions but also cell volume regulation, all of which impact intracellular vacuole dynamics (26). Once the aBCV reaches a given size, ranging from 15 to 650 μm^3^, the cell is compelled to release the bacterial clusters. However, larger BECs above 250 μm^3^ are rare, suggesting that the process of shedding smaller BECs (e.g., <100 µm^3^) is constitutively sustained.

The close packing of bacteria within BECs suggests that the *Brucella* coccobacilli follow the rules of the closest-packed configuration, which depends on the number and size of bacteria (16). At least for less than 50 brucellae coccobacilli, the packing structures are arranged in polyhedrons of various shapes. For instance, the packing of 12 coccobacilli approximates a cube (16). In contrast, the packing of 50 and 100 coccobacilli approximates spherical configurations. Nevertheless, the packing structures significantly differ. Although larger BECs may maintain a spherical shape, their bacterial arrangements will vary, depending on the vacuole size (16). Higher numbers of bacteria are predicted to make the BECs less stable and prone to disruption. Indeed, the aBCVs and BECs, with more than 250 bacteria, are less abundant than those with fewer bacteria.

Despite being ordered within BECs, there is bacterial heterogeneity regarding dead and live organisms, as well as the metabolic activity of the former. Although most live bacteria inside BECs remain metabolically active, a small proportion are inactive, and nearly ~18% to 25% are dead bacteria. While metabolically live bacteria enhance their ability to infect other cells, dead bacteria are likely donors of cohesive material, such as DNA and histone-like proteins (e.g., MucR), potentially acting as “glue” for bacterial aggregation (27, 28). This tight clumping probably forms microcolonies that shield against bactericidal agents and facilitate quorum sensing for initiating a new infection cycle (29).

From the standpoint of composition (LAMP-1, actin filaments, acidity, and size), aBCVs and BECs display very similar properties, and it is well known that intracellular acidity triggers the expression of genes necessary for the bacteria’s survival and replication inside host cells (30). Indeed, *Brucella* aBCVs’ autophagic characteristics may be related to mechanisms that activate virulent processes, enhancing bacterial infectivity and adhesion as described previously (30). Moreover, bacteria isolated directly from infected cells at later stages exhibit enhanced virulence, as demonstrated by higher adherence and penetration capabilities, compared to those grown in bacteriological cultures (30). This phenomenon is linked to the BvrR/BvrS system, which recruits VjbR and VirB virulence factors (30).

It is not known whether brucellae within the aBCVs promote their exit through type IV bacterial effectors that eventually trigger cytoskeletal polymerization through the action of small GTPases. It was observed that heavily infected cells produce cytoplasmic protrusions accompanied by the polymerization of cytoskeletal actin proteins, resulting from the persistent local recruitment of small GTPases of the Rho subfamily. The fact that LAMP-1 and actin remain closely associated with BECs indicates the ubiquity of this process during the *B. abortus* intracellular biogenesis. The actin-rich filopodia-like protrusions may also facilitate the intercellular bacterial infection (31), a phenomenon that we have concurrently observed in *B. abortus*-infected cells (32). Analogous to the initial activation of small GTPases during the entry of invading *Brucella* organisms into HeLa cells (4), the exit o*f B. abortus* is also promoted by the participation of GTPases of the Rho subfamily. The activation of Rho GTPases, which control the dynamics of the cytoskeleton, could be a ubiquitous mechanism for the release of large cell aggregates, as they are also required for the protrusion of apoptotic bodies (33), a phenomenon reminiscent of the release of BECs. Although further studies are needed to determine the precise spatial recruitment of actin and the activation of Rho GTPases, our results using specific activators and inhibitors demonstrate that host cell cytoskeletal integrity is a key factor in the egress mechanism

Previous studies show that vacuolar lytic enzymes intracellularly degrade autophagic bodies, and the degradation products are reused or released (34, 35). However, this is not true for the aBCVs, which exhibit autophagic characteristics (8). In *Chlamydia trachomatis* intracellular parasitism, membrane-bound bacterial clusters budding from the cells are released through the concerted action of GTPases of the Rho subfamily and the concomitant recruitment of actin filaments (20). As in *Chlamydia*, the generation of cytoskeletal filaments in *Brucella*-infected cells, associated with the formation of cytoplasmic extensions, is also linked with the BEC protrusion. However, chlamydial infection vesicles lack autophagosome characteristics, are not acidic, and do not contain LAMP-1; instead, they display properties of early endosomes (36).

Intracellular *B. abortus* organisms within neutrophils also promote their infectivity toward macrophages that ingest these infected polymorphonuclear cells. Once inside macrophages, the *Brucella* organisms replicate significantly more than when macrophages are infected with bacteria alone (37, 38). Like *Brucella*-infected neutrophils, the BECs are internalized by other cells and initiate a new infection cycle with a substantial bacterial load. As in neutrophils, these bacteria also seem highly virulent (30, 37).

The ability of a proportion of *B. abortus* to exit encased within a host cell-derived membrane through mechanisms that do not involve cell death provides significant advantages. Indeed, the acidic membrane-bound BECs with the characteristics of autophagosomes or apoptotic bodies may be internalized by professional phagocytes, such as macrophages, neutrophils, or dendritic cells, thereby suppressing proinflammatory signals (39–41). In contrast to naked bacteria, readily recognized by the immune system, membrane-bound BECs may evade immune detection. It remains to be solved whether the membrane-bound BECs are more infectious than the naked ones or vice versa. It is plausible that the naked BECs are more active in infecting neighboring cells, while the membrane-bound BECs travel larger distances, evading antibodies, complement, or other bactericidal substances in the extracellular milieu. These phenomena would enable *Brucella* organisms to maintain their stealthy strategy, establish a persistent infection, and facilitate dissemination to other cells and hosts (15). It is also possible that a small percentage of clusters may lose their membrane due to mechanical stress during the protocol. While these structures suggest the presence of non-membranous bacterial aggregates, we cannot formally exclude the possibility that the absence of a surrounding host-derived membrane may result from technical artifacts during sample processing for electron microscopy, such as membrane stripping during fixation or dehydration.

## MATERIALS AND METHODS

### Bacterial strains and culture

*B. abortus* 2308, expressing a constitutive RFP from *Discosoma* coral (*B. abortus*-RFP) (Unité de Recherche en Biologie Moléculaire, Facultés Universitaires Notre-Dame de la Paix, Namur, Belgium), *B. abortus* 2308W, expressing a constitutive green fluorescent protein (*B. abortus*-GFP) (41, 42), and *B. abortus* 2308W with the ATc-inducible pJC45-gfp plasmid (*B. abortus* 2308W-pJC45-gfp) were used in this study (43). In addition, *B. abortus*-RFP was electroporated with the ATc-inducible pJC45-gfp plasmid (*B. abortus*-RFP-pJC45-gfp) following previous protocols (43). *Brucella* strains were grown on trypticase soy agar (TSA), inoculated in 20 mL of trypticase soy broth (TSB), and incubated at 37°C with constant shaking for 24 h. Colony-forming units per milliliter were estimated by the absorbance of the culture in TSB at 420 nm and measured using a previously standardized formula (30). All the *B. abortus* strains used displayed a similar replicating pattern in cells and were fully virulent.

### Infection of cell cultures

HeLa ATCC clone CCL-2 cells were cultured and infected with *B. abortus* 2308W-pJC45-gfp, *B. abortus*-RFP, *B. abortus*-RFP-pJC45-gfp, or *B. abortus*-GFP as described previously (30). Briefly, for fluorescence analysis, HeLa cells were seeded on 13 mm glass slides in 24-well tissue culture plates 2 days before infection to obtain a final concentration of 5 × 10^5^ cells per well. For live imaging, HeLa cells were seeded in 96-well culture plates 2 days before infection to obtain a final concentration of 3 × 10^4^ cells per well prior to analysis using a BioTek Lionheart FX Automated Microscope (Agilent). A multiplicity of infection of 500 was determined by estimating the bacterial population using optical density, followed by diluting the bacteria in Dulbecco’s modified Eagle medium (DMEM) supplemented with 5% fetal bovine serum. Infected cells were centrifuged at 330 *× g* for 5 min at 4°C and incubated at 37°C and 5% CO_2_ for 45 min. Each well was washed in triplicate with 1× PBS and incubated for 1 h with DMEM medium supplemented with 100 μg/mL gentamicin to remove extracellular bacteria. The medium was then replaced with DMEM maintenance medium supplemented with 5 μg/mL gentamicin and incubated for the indicated hours in each assay. Gentamicin was initially removed 4 h prior to the indicated assays to ensure the recovery of all viable extracellular bacteria. However, subsequent experiments confirmed that the membrane-bound BECs remain resistant regardless of the presence of the antibiotic, and the gentamicin was not removed.

### Intracellular and extracellular bacterial quantitation

Quantitation of intracellular and extracellular *B. abortus* organisms was performed as described before (30). Briefly, the DMEM maintenance medium supplemented with 5 μg/mL gentamicin in the cell culture was discarded and replaced with DMEM without gentamicin. After incubation for 4 h, the cell supernatants were taken, and serial dilutions were performed and plated in tryptic soy agar (TSA). Concurrently, intracellular bacteria were determined at the same intervals. The infected cells were disrupted with a 0.1% Triton X-100 solution for 10 min. Quantitation was performed either by direct counting of red fluorescent bacteria under the microscope or through dilutions followed by plating on TSA and culturing for 48 h. This procedure was repeated 6, 24, 48, 54, 66, 72, and 96 h

### Enrichment of aBCVs and BECs

HeLa cells seeded in a six-well plate were infected, and at 48 h p.i., the cell culture maintenance medium was changed to a medium without gentamicin to allow BECs to exit from infected cells. The supernatant with BECs and free bacteria was then collected. Thereafter, the infected cells were washed with DMEM and lysed with 0.1% Triton X-100 in 1× PBS, and the suspension was collected after 10 min of incubation. Supernatants and cell lysates were used to enrich aBCVs and BECs, using the same protocol. The suspensions were centrifuged at room temperature at 90 *× g* for 5 min. The resulting supernatants were centrifuged at 300 *× g* for 5 min at room temperature. The sediment obtained from the second centrifugation, containing purified aBCVs or BECs, was processed for analysis using fluorescence or electron microscopy, as described below.

### Fluorescence microscopy

Fluorescence microscopy was performed as described previously (4, 30). *B. abortus*-infected HeLa cells or enriched vesicles were fixed with ice-cold 3% paraformaldehyde for 10 min. Then, cells and vesicles were washed in 1× PBS and incubated for 10 min with 1× PBS containing 50 mM NH_4_Cl. To stain DNA in cell nuclei and bacteria, infected cells were incubated for 10 min with 1× PBS containing 0.5 µg/mL diamidino-2-phenylindole (DAPI) (Thermo Fisher Scientific). Samples were processed with different treatments, depending on the experiment (as indicated in the figures). Cells or vacuoles were stained with 6.6 µM Alexa Fluor 488-phalloidin (Invitrogen) in a 10% horse serum solution for 30 min to detect actin. aBCVs and BECs were stained with a 0.5 µg/mL wheat-germ agglutinin conjugated with Alexa Fluor 488 (WGA-AlexaF-488; Invitrogen). aBCVs and BECs were labeled with a 1 µg/mL LAMP1 rabbit monoclonal antibody H4A3 (Abcam) for 30 min and 2 µg/mL Alexa Fluor 488 anti-rabbit conjugate (Invitrogen) for 30 min. To determine the acidity of the protruding BECs, infected HeLa cells were incubated with 50 nM LysoTracker Red DND-99 (Invitrogen) at 48 h post-infection for 1 h before analysis.

We followed a previously described procedure to determine if aBCVs and BECs are enclosed within an impermeable host membrane (30). Briefly, to label bacteria found on membraneless vesicles, BECs and aBCVs cells were washed three times with 1× PBS and incubated for 30 min at 4°C with an in-house cow IgG conjugated with FITC, mostly reacting against the *Brucella* smooth LPS, as evaluated by Western blot (13). Cells were then fixed with ice-cold 3% paraformaldehyde for 10 min. Samples were washed once, incubated for 10 min with 1× PBS containing 50 mM NH_4_Cl, and permeabilized for 10 min with 0.1% Triton X-100. After incubation, the slides were washed three times with 0.1% Triton X-100, and the nuclei were stained with 0.5 μg/mL DAPI (Thermo Fisher Scientific). Finally, slides were mounted using ProLong Gold Antifade Mountant (Invitrogen) before analysis by fluorescence microscopy. Before and after each experiment, cells were examined with 1 µg/mL Hoechst (Abcam) to preclude *Mycoplasma* contamination.

For live imaging analysis, infected cells, aBCVs, and BECs were recorded using a BioTek Lionheart FX Automated Microscope for up to 100 h. Confocal imaging was performed with a Nikon A1R HD25 laser-scanning microscope equipped with ×60 (Plan Apo λ Oil) and ×100 (SR HP Apo TIRF) objectives. The microscope was equipped with a Nikon A1 LFOV camera and four laser lines at 405 nm (20 mW), 488 nm (70 mW), 561 nm (70 mW), and 647 nm (125 mW), with the following emission/excitation wavelengths: DAPI 450/405 nm, FITC 525/488 nm, and TRITC 595/561 nm. Images were acquired with NIS-Elements Advanced Workstation Software 5.42.04 (Nikon). All images were captured in 12-bit format with a resolution of 1,024 × 1,024 pixels at room temperature. When stated, raw images were processed using the denoise.ai module.

### Size determination of aBCVs and BECs

The enriched, fixed, and stained aBCVs and BECs were observed under a confocal fluorescence microscope using the above-described software. The diameters of the vesicles were measured, and their volume was estimated. Likewise, the average sizes and volume of *B. abortus* were determined by measuring bacteria in photographs taken through transmission and scanning microscopy. Based on these measurements, the average volume of *B. abortus* coccobacilli was estimated to be 0.49 ± 0.04 µm^3^. The number of bacteria in each vesicle was estimated using the formula: vesicle volume µm³/ *B. abortus* average volume µm³ × 0.74 (the estimated proportion of space filled by rods packed within a sphere). For the estimation of the packing order of bacteria within BECs, photographs of the vesicles were amplified, and the number of fluorescent spots was counted using the Adobe Photoshop density color pixel function of the image’s sections, and the method for the close packing of rods on a spherical surface was followed (16).

### Determination of live, dead, and metabolically active *B. abortus*

*B. abortus*-GFP, *B. abortus* 2308W-pJC45-*gfp*, and *B. abortus*-RFP-pJC45-*gfp* were used to evaluate live and dead bacteria and protein synthesis in “metabolically” active bacteria. After the purification of aBCVs and BECs, GFP expression of *B. abortus*-pJC45*-gfp* or *B. abortus*-RFP-pJC45-*gfp* was induced by adding 250 nM anhydrotetracycline for 6 h. *B. abortus*-RFP-pJC45-*gfp*, displaying red and green fluorescence, was considered alive and metabolically active in protein synthesis. Likewise, *B. abortus* 2308W-pJC45-*gfp*, displaying green fluorescence, was considered metabolically active in protein synthesis. *B. abortus*-RFP-pJC45-*gfp*, displaying only red fluorescence, was considered alive but metabolically inactive. Bacteria stained with 1× PBS containing 1 µg/mL propidium iodide were recorded as dead bacteria (17). All bacteria (dead and alive) were stained blue with 1× PBS containing 0.5 µg/mL DAPI. For some experiments, the enriched aBCVs and BECs were disrupted with 0.1% Triton X-100 dissolved in 1× PBS before counting the bacteria under the fluorescent microscope. The ratio of red fluorescence versus green fluorescence per bacteria was estimated by using the histogram function of the Adobe Photoshop program of 50 single *B. abortus*-RFP-pJC45-*gfp* expressing or not expressing GFP protein (17). Due to the higher intensity of the RFP signal, which partially masked the GFP fluorescence in merged images, the red and green ratio was calculated. This normalization ensures a more accurate visualization and interpretation of the protein distribution, independent of the differences in fluorochrome emission strength.

### Analysis of cells, aBCVs, and BECs by electron microscopy

The pellets obtained from infected HeLa cells were fixed with a modified Karnovsky solution (2% glutaraldehyde and 2% paraformaldehyde in 0.05 M phosphate buffer, pH 7.2, for 2 h) and then washed with phosphate buffer. Then, they were post-fixed with 1% osmium tetroxide fixative and washed with distilled water. Subsequently, the sample was dehydrated using ethanol gradients (30%, 50%, 70%, 80%, 90%, 95%, and 100%) for 15 min. Samples were dried, and finally, the slides with infected cells were mounted on an aluminum base and recovered with gold (12 nm) in a Sputter coater Denton Vacuum model DESK V (Denton Vacuum, Inc.). Cells were analyzed using an electron scanner JEOL JSM-6390 LV with an acceleration voltage between 10 and 20 kV, and images were taken at different magnifications.

Transmission electron microscopy was performed as described previously, with slight modifications (4). Briefly, *B. abortus* 2308W-infected HeLa monolayers were fixed with 2.5% glutaraldehyde and 2% paraformaldehyde in 0.1 M phosphate buffer. Fixed cells were incubated at 40°C for 5 min, and 100 μL of 3% low-melting agarose at 40°C was added. The temperature was lowered, and 5 vol of 2.5% glutaraldehyde in PBS was added to the solid agarose block and incubated overnight at 4°C. Samples were placed in 1% OsO_4_ solution for 1 h post-fixation, dehydrated in a graded ethanol concentration, and infiltrated with Spurr resin. Thin sections on 300-mesh collodion-coated grids were stained with uranyl acetate and lead Sato’s solution. Preparations were examined with a Hitachi H-7100 electron microscope operating at 75 kV.

### Participation of small GTPases of the Rho subfamily

Three nanograms per milliliter of CNF 1 or 1 µg/mL cytochalasin B was added to infected cells at 48 h p.i. for 2 h. Cells were washed with 1× PBS and at 72 h p.i., fixed with paraformaldehyde or modified Karnovsky solution, and prepared for fluorescence or electron microscopy analysis as previously described. Wells without CNF or cytochalasin B were used as controls. Additionally, at 72 h p.i., the DMEM maintenance medium, supplemented with 5 μg/mL of gentamicin, was changed to a gentamicin-free medium to promote bacterial egress from infected cells. After 4 h of incubation, supernatants from infected cells were transferred, serially diluted, and plated in TSA. After washing the infected cells with 1× PBS, the cells were lysed with 0.1% Triton X-100 for 10 min. The supernatant was then diluted, and bacteria were counted by plating them in TSA and culturing for 48 h at 37°C, as described previously (30).

For live imaging analysis, 3 ng/mL CNF 1 or 50 ng/mL *Clostridioides difficile* toxin B (TcdB) was added to infected cells at 48 h p.i., for 2 or 1 h, respectively. Cells were washed with 1× PBS and aBCV and BECs were recorded using a BioTek Lionheart FX Automated Microscope for up to 100 h as described above. Wells without CNF or TcdB were used as controls.

### BEC infection assessment

The maintenance medium with gentamicin from infected HeLa cells was changed to DMEM medium without gentamicin or maintained with gentamicin at 48 h p.i. The proportion of newly infected cells through released BECs was assessed through live imaging using a BioTek Lionheart FX Automated Microscope. Cell nuclei were stained with DAPI as described above.

### Immunochemical analysis

To analyze the LPS composition of the purified aBCV and BECs, samples were lysed with 2% SDS and run on SDS-PAGE and Western blot. For Western blot analysis, the primary antibodies M84 (-Br-Mouse LPS) and YST9 (-Br-LPS HRP-conjugate) (44) were used to detect Br-LPS in the lysates of BCVs.

### Statistical analysis

Student *t*-test and Kruskal-Wallis and Dunn multiple-comparison tests were used accordingly. The significance was indicated as *, *P* < 0.05; and **, *P* < 0.005.

## SUPPLEMENTAL MATERIAL

Fig S1 msphere.00450-26-s0001.tif BECs protrude by macrophage cells and initiate a new cycle of infection.

Fig S2 msphere.00450-26-s0002.tif Validation of *Brucella* LPS structure and BEC membrane impermeability.

Video S1 msphere.00450-26-s0003.mp4 *Brucella* exiting from cells.

ASM does not own the copyrights to Supplemental Material that may be linked to, or accessed through, an article. The authors have granted ASM a non-exclusive, world-wide license to publish the Supplemental Material files. Please contact the corresponding author directly for reuse.
